# Recent Advances in Mechanochromism of Metal-Organic Compounds

**DOI:** 10.3389/fchem.2022.865198

**Published:** 2022-03-04

**Authors:** Xiao-Yan Wang, Liqiang Lv, Li Sun, Yue Hou, Zhenghao Hou, Zhao Chen

**Affiliations:** ^1^ College of Chemical Engineering, Shijiazhuang University, Shijiazhuang, China; ^2^ Jiangxi Key Laboratory of Organic Chemistry, Jiangxi Science and Technology Normal University, Nanchang, China

**Keywords:** mechanochromism, complex, metalophilic interactions, mechanism, phase transition

## Abstract

Smart luminescent materials, which can respond to the changing of external environment (light, electricity, force, temperature, etc.), have always been one of the research hotspots. Mechanochromism refers to the materials whose emission color or intensity can be altered under the stimulation of external mechanical force. This kind of smart materials have been widely used in data storage, information encryption and sensors due to its simple operation, obvious and rapid response. The introduction of metal atoms in metal-organic compounds brings about fascinating metalophilic interactions and results in more interesting and surprising mechanochromic behaviors. In this mini-review, recent advances in mechanochromism of metal-organic compounds, including mono-, di-, multinuclear metal-organic complexes and metallic clusters are summarized. Varies mechanisms are discussed and some design strategies for metal-organic compounds with mechanochromism are also presented.

## Introduction

**FIGURE 1:**
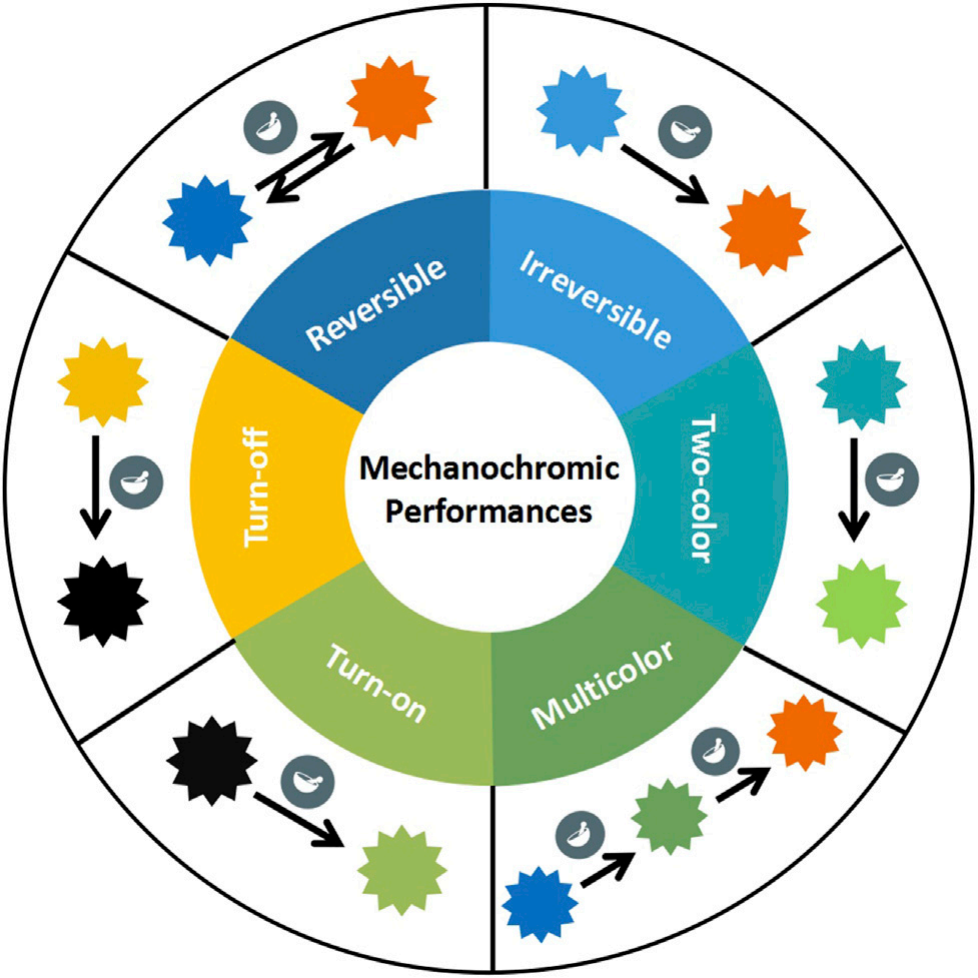
Schematic of mechanochromic performances.

Luminescent materials have always been valued by researchers due to their widely use in data storage, optoelectronic devices, biological imaging and other fields (Zhu et al., 2016; Shi et al., 2018; Wang et al., 2021; Jackson et al., 2021; Yin et al., 2021; Min et al., 2022). Metal-organic compounds (MOC), composed of metal ions and organic ligands, are expected to exhibit more interesting photophysical properties. Generally, metal-organic compounds are more conducive in practical application due to their long-lived triplet excited lifetimes and large Stokes shifts. The luminescence color of MOC can be regulated by changing the ligands or by external stimuli, such as light, electricity, temperature, mechanical force, etc (Takeda et al., 2020; Yang M et al., 2020; Yang W et al., 2020). Mechanochromism, a subclass of smart stimuli-responsive materials, refers to the solid materials whose luminescent color or intensity can be changed under the stimulus of mechanical force. Mechanochromic materials have been widely used in data storage, information encryption and sensors due to their simple operation, obvious and fast response (Perruchas et al., 2010; Zhang et al., 2017; Seki et al., 2017; Ito et al., 2008; Song et al., 2018; Li et al., 2019; Zhu et al., 2020). The emission color of mechanochromic materials usually have wide luminous range, covering the entire visible region and even reaching in the near-infrared region (Seki et al., 2017; Zhang et al., 2021; Li et al., 2021). Based on their different phenomenons, mechanochromic materials can be divided into various classes (Figure 1): reversible and irreversible mechanochromism, two-color and multicolor mechanochromism, turn-on and turn-off typed mechanochromism (Yang et al., 2018; Liu et al., 2019; Yang W et al., 2020; Fan et al., 2021; Qian et al., 2021).

**FIGURE 2:**
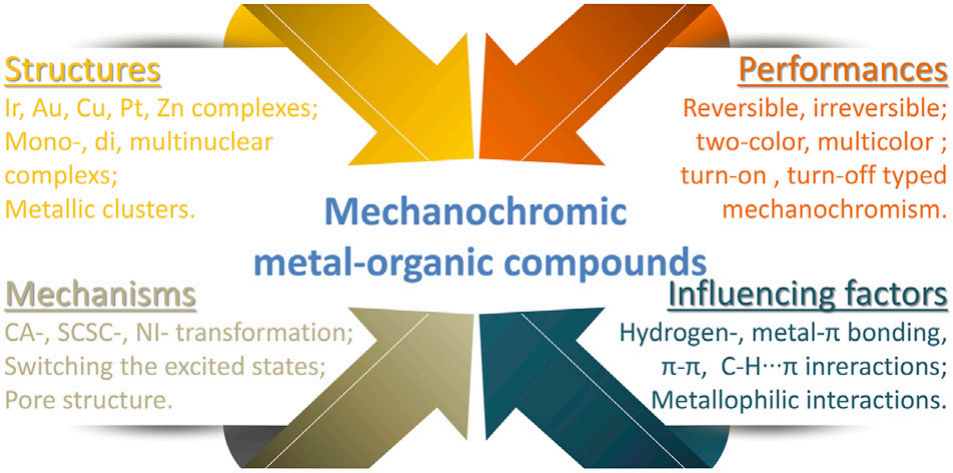
Mechanochromism of metal-organic compounds.

To date, most of the reported mechanochromic materials are based on organic molecules (Huang et al., 2019; Jiang et al., 2019; Li et al., 2020; Li and Li, 2020). Traditional organic mechanochromic materials often rely on the change of molecular packing patterns, intermolecular π-π interactions and inermolecular hydrogen-bonding interactions. Although the current research on mechanochromic metal-organic compounds is limited, such mechanochromic materials are still attractive and are expected to exhibit more interesting phenomena due to the metalophilic interactions existed in metal complexes. These mechanochromic phenomenons are thought to result from the crystalline-to-amorphous (CA) phase transformation, single-crystal-to-single-crystal (SCSC) phase transformation or triplet excited states transformation caused by the intermolecular conformational folding or twisting, as well as changes in intermolecular π-π, metalophilic or hydrogen-bonding interactions (Seki et al., 2013; Ito et al., 2013; Seki et al., 2015; Jin et al., 2017; Jin et al., 2018; Li et al., 2019; Pei et al., 2020; Xie et al., 2020). Indeed, an explicit mechanism has not been investigated yet because of the difficulty in characterizing the amorphous phase. Furthermore, the relationship between molecular structures and mechanochromic properties has not been systematically studied, which makes it difficult to find an efficient and simple design strategy for mechanochromic complexes. In this mini-review, a comprehensive summary of recent advances in mechanochromic metal-organic compounds was provided (Figure 2). According to the number of metal atoms in the compounds, we summarized the mono-, di-, and multinuclear complexes, even metallic clusters (Benito et al., 2015; Li et al., 2017; Zhang et al., 2017; Ma, 2019) and metal-organic framework (MOF) (Wei et al., 2016; Wu and Huang, 2018; Jindal et al., 2021) with mechanochromisms, including iridium, platinum, gold, copper and zinc etc. At the same time, the different mechanochromic mechanisms of these complexes were also discussed. Through this review, effective methods for further designing mechanochromic materials based on metal complexes are expected to provide.

## Mechanochromism of Iridium Metal-Organic Compounds

### Mononuclear Ir(III) Complexes With Mechanochromism From CA Transition

Intermolecular interactions play a vital role in mechanochromic materials. Introducing halogen, or oxygen-containing functional groups into ligands is an important way to obtain mechanochromic complexes. Based on this strategy, R. Bryce et al. rationally designed a neutral iridium (III) complex with reversible mechanochromism (Li et al., 2019). The carboxyl and electron-accepting F substituents were incorporated to form intermolecular interactions such as π-π interactions, C-H‧‧‧F and O-H‧‧‧O hydrogen bonds, which played a vital role to achieve the mechanochromism of the iridium (III) complex. Both fourier transform infrared spectroscopy (FTIR) and X-ray photoelectron spectroscopy (XPS) indicated that these intermolecular interactions were broken under grinding. The change of emission color from orange to yellow was resulted from CA transition. From this study, introducing multi-interactions is an effective and facile design strategy to obtain mechanochromic Ir(III) complexes.

Apart from traditional interactions, some infrequent interactions can also promote the formation of loose packing modes. Under mechanical stimulation, the change of interactions lead to the change of stacking modes, resulting in the mechanochromism. Pei and Zhao’ group prepared a series of cationic Ir(III) complexes with significant mechanochromic luminescence (Pei et al., 2020; Zhao et al., 2021). For the first time, the hydroxyl substituents were introduced into Ir(III) complexes to form π-hydrogen bonding. The intermolecular interactions can be easily destroyed by external stimulation, leading to the effective change of luminescent color and showing mechanochromism. Loose packing mode formed by π-hydrogen bonding is critical to establish stimuli responsive phosphorescent materials for Ir(III) complexes.

### Dinuclear Ir(III) Complexes With Mechanochromism From CA Transition

Compared with the mononuclear complexes, more functional groups can be introduced into the dinuclear metal complexes, and more excellent luminescence properties can be obtained in the dinuclear complexes (Xie et al., 2020). For mechanochromic dinuclear Ir(III) complexes, CA phase transition was still the main mechanism. In recent years, researchers have proposed several specific methods to achieve the stimuli-response properties of iridium complexes. On the one hand, AIE-active units such as tetraphenyl ethylene (TPE) were introduced into the Ir(III) complexes to enhance the luminescent properties in solid states (Li et al., 2020). On the other hand, conformationally flexible Schiff base ligands such as acylhydrazones were introduced into the complexes to enhance intermolecular hydrogen-bonding interactions in the crystals (Jiang et al., 2017a; Jiang et al., 2017b; Li et al., 2020; Xie et al., 2020). In conclusion, both AIE performance and hydrogen-bonding interactions are important for the construction of mechanochromic dinuclear Ir(III) complexes.

## Mechanochromism of Gold Metal-Organic Compounds

### Mononuclear Au(I) Complexes With Mechanochromism From CA Transition

The most common mechanism for mechanochromism of complexes was the CA phase transition. Seki et al. reported a number mononuclear Au(I) complexes with mechanochromism, whose emission colors could be red-shifted or blue-shifted (Seki et al., 2018). In 2017, Seki s group prepared a 9-anthryl gold(I) isocyanide complex, which exhibited a bathochromic shift of its emission color from the visible (448 nm) to the infrared (IR) region (710 nm) (Seki et al., 2017). The results of the study indicated that intramolecular ligand-to-ligand charge transfer (^3^LLCT) was existed in the original crystallic solids of gold(I) complexes, and the aurophilic interactions were formed after grinding, resulting to the formation of metal-metal-to-ligand charge transfer (^3^MMLCT) states and the extension of the π system, which reduced the energy level of the luminophore and caused red-shifted emission. Notably, this complex possessed the largest red-shifted emission reported so far and provided a facile method to construct infrared light-emitting materials by mechanochromism. On the contrary, some blue-shifted mechanochromic gold(I) complexes were also found. An arylgold (arylisocyanide) complex with hypsochromic mechanochromism was reported in 2018 (Seki et al., 2018). Strong aurophilic interactions were found in the original solids of this complex. However, the weaker aurophilic interactions were detected under the stimulus of mechanical force, which lead to the blue-shifted emission.

### Dinuclear Au(I) Complexes With Mechanochromism From CA Transition

A few dinuclear Au(I) complexes with mechanochromism have been reported (Jobbágy et al., 2014a; Jobbágy et al., 2014b; Deák et al., 2015). In 2002, Ito’s group reported the first dinuclear Au(I) complex with reversible red-shift mechanochromic properties (Ito et al., 2008) resulting from CA phase transition. In 2015 and 2016, Chen et al. designed a series dinuclear Au(I) complexes with AIE and mechanochromic properties (Chen et al., 2015; Chen et al., 2016). Similarly, the generation of aurophilic interactions after grinding could turn on or change the luminescence of Au(I) complexes. And the mechanochromism of these complexes were resulted from CA phase transition.

### Mononuclear Au(I) Complexes With Mechanochromism From SCSC Transition

Apart from CA transition, mechano-induced single-crystal-to-single-crystal (SCSC) phase transition was another important mechanism for mechanochromic gold(I) complexes (Ito et al., 2013; Seki et al., 2013; Seki et al., 2015; Jin et al., 2017; Jin et al., 2018). The phase transfer processes could be monitored clearly: The change first happened at the point of contact and then spontaneously spread throughout the entire crystal. Further research indicated that the transition of the intermolecular interactions from C-H‧‧‧π to aurophilic interaction was accompanied by the SCSC phase transition.

### Au(I) Complexes With Mechanochromism From NI Transition

The researchers were surprised to find that the neutral-to-ionic (NI) phase transition could contribute to the mechanochromism of Au(I) complexes. In 2015, Baranyai’s group reported a gold(I)-diphosphine complex with reversible mechanochromism (Baranyai et al., 2015). The original neutral mononuclear solid emitted blue luminescence. After mechanical grinding, the neutral mononuclear complex was converted to a ionic dinuclear structure with intramolecular aurophilic interactions, emitting red photoluminescence and showing high contrast mechanochromism.

### Mononuclear Au(I) Complexes With Multicolor Mechanochromism

Both CA and SCSC transitions occured between two phases and caused bicolor mechanochromism. The existence of another metastable state enabled the complexes to exhibit multicolor mechanochromic properties (Zhang Y et al., 2015; Takeda et al., 2018). Wang et al. designed a gold(I)-isocyanide complex demonstrating interesting tricolor mechanochromism feature (Wang et al., 2018). Its emission colors were changed from blue to cyan upon slight grinding, and the cyan light-emitting solids were converted into orange solids after heavy grinding. Interestingly, this orange metastable state could be recovered to the original blue state without any treatment, exhibiting self-recovery tricolor mechanochromism.

## Mechanochromism of Copper Metal-Organic Compounds

### Mononuclear Cu(I) Complexes With Mechanochromism From Cu−F Interactions

The photoluminescence properties of Cu(I) complexes are greatly affected by cuprophilic interaction. However, it is difficult to predict the formation or destruction of Cu-Cu interactions, so it is urgent to develop a simple method to construct mechanochromic copper complexes. Liske’ group investigated some Cu(I) complexes and came up with a new mechanism for mechanochromism (Hupp et al., 2018; Liske et al., 2019). They thought that enhancing the interactions between the cationic metal complexes and suitable counterions could shorten the distance of cation-anion contact and prompt the formation of Cu‧‧‧F interactions, which resulted in the mechanochromic luminescence of Cu(I) complexes. Indeed, this might be a general design strategy worth pursuing.

### Mononuclear Cu(I) Complexes With Mechanochromism From the Pore Structure

In the aforementioned Cu(I) complexes, the emission colors could not be returned the original states, showing irreversible mechanochromism. In order to obtain reversible mechanochromic materials based on Cu(I) complexes, flexible and rigid ligands were introduced to prompt the formation of loose stacking modes. Yu et al. reported a steric bis(pyrazol-1-yl)borohydrate-containing Cu(I) complex with reversible mechanochromism (Yu et al., 2021). The study indicated that the pore structure existed in the crystal provided a flexible and loose space, the coordination environment was changed under the stimuli of mechanical force, leading to the distinct mechanochromism.

### Dinuclear Cu(I) Complexes With Mechanochromism From Cuprophilic Interaction

Similar to other complexes, cuprophilic interaction is important factor for mechanochromic dinuclear Cu(I) complexes. It is crucial to discuss the relationship between structural changes such as Cu-Cu distance and luminescence performance. In 2018, the mechanochromisms of dinuclear Cu(I) complexes bearing N-heterocyclic carbene (NHC) ligands were studied by Lu’s group (Lu et al., 2018). The stimulus-response performance of these complexes came from the CA phase transition. Detailedly, the crystal lattice and the coordination environment of Cu center were destroyed by the stimuli of mechanical force, leading to the shortening of the Cu-Cu distance and the change in emission color. In addition, introducing nitrogenous ligands into the dinuclear Cu(I) complexes was also beneficial to construct mechanochromic Cu(I) complexes. For example, two N-H groups were introduced into the ionic bimetallic Cu(I) complexes to form the N−H∙∙∙O hydrogen bonding with the neighbouring ClO_4_^−^ anion (Chen et al., 2020). The destruction and recovery of the hydrogen bonding resulted in their reversible mechanochromism.

### Multinuclear Cu(I) Complexes With Mechanochromism

In recent years, a few trinuclear or tetranuclear Cu(I) complexes with mechanochromic properties resulting from CA phase transition have been reported successively. Xie et al. reported a carbazole-based cyclic trinuclear Cu(I) complex with abnormal pressure-induced phosphorescence enhancement (PIPE) (Xie et al., 2021). The research results indicated that the weak π-acid‧‧‧base interactions existed between the Cu unit and the carbazole group and the Cu‧‧‧Cu interactions played an important role for their turn-on mechanochromism. For tetranuclear Cu(I) complexes, there were many factors that might affect the mechanochromism, such as the change of the Cu‧‧‧Cu distance, π-π interactions and the complex geometry (Benito et al., 2014; Peng et al., 2020; Utrera-Melero et al., 2020; Perruchas, 2021). In 2020, Hu’s group discovered a supersalt-type Cu(I)-thiolate cluster with irreversible mechanochromism for the first time, which was not completely caused by the CA phase transition (Hu et al., 2020). Surprisingly, only the stacking mode was changed and the crystallinity was only destroyed a little. In 2018, a series of luminescent coordination polymers composed of copper(I) iodide were found by Kobayashi’s group (Kobayashi et al., 2018). The switching from thermally activated delayed fluorescence (TADF) to phosphorescene was realized by the stimulus of force, which was caused by the change of excited states from triplet metal-to-ligand and halide-to-ligand charge-transfer (^3^ML/^3^HLCT) excited states to cluster-centred (^3^CC) excited states. In 2017, Xin’s group found a pillared-layered copper(I) MOF, whose mechanochromic performance was resulted from the weak bond contraction of the copper halide layer (Xin et al., 2018). Apart from cuprophilic interaction, the alteration of π‧‧‧π interactions also could give rise to the mechanochromism of Cu MOF (Zhang DX et al., 2015).

## Mechanochromism of Other Metal-Organic Compounds

### Pt(II) Complexes With Mechanochromism From Switching the Triplet Excited States

Pt (II) complexes have unique photophysical properties due to their multiple excited states, such as LE (local excitation), MLCT (metal-to-ligand charge transfer), LLCT (ligand-to-ligand charge transfer), and ILCT (intraligand charge transfer). These excited states would be changed under the stimuli of force, showing mechanochromism. In 2020, Zhu et al. reported Pt (II) complexes with mechanochromic properties (Zhu et al., 2020). The emission wavelength of original solid powders was at 541 nm, showing orange phosphorescence; a bathochromic red phosphorescence was observed at 639 nm after grinding. Density functional theory (DFT) calculations indicated that the mechanochromic behavior of this complex might be caused by the change in the intermolecular Pt-Pt distance, which resulted to a switching emission state from ^3^π-π*/^3^MLCT to ^3^MLCT. This example proposed a new mechanism of mechanochromic properties and provided a new design strategy to tune phosphorescence by switching triplet excited states.

### Zn(II) Complexes With Mechanochromism From CA Transition

Zinc is abundant and inexpensive compared with other noble metals, so it is more attractive to design zinc complexes with mechanochromic properties, which would facilitate the development of low-cost sensor materials. In 2018, Song et al. designed a Zn(II) schiff-based complex (Song et al., 2018). The complex displayed high-contrast mechanochromism. XRD, fluorescence microscopy and SEM images demonstrated that the emission-colour transition was caused by the change of molecular packing modes and CA transition.

## Summary and Outlook

This paper reviewed the recent advances in mechanochromism of metal-organic compounds such as Ir(III), Au(I), Cu(I), Pt (II) and Zn(II) complexes. Considering the structures of complexes, mono-, di-, multi-nuclear complexes and even clusters, metal-organic frameworks (MOF), and coordination polymers have been summarized according to the type and number of metal atoms. Considering the mechanochromic properties of complexes, various mechanisms were outlined: CA and SCSC phase transitions, NI transition, the pore structure and switching the triplet excited states. By exploring the structure-property relationship of these complexes, several effective design strategies have been proposed: 1) Introduce heteroatom-containing groups to form multiple intermolecular interactions especially the metallophilic interactions; 2) Introduce large flexible groups to form the loose packing modes; 3) Construct the AIE complexes. Despite this, mechanochromic materials still lacked a clear theory because of the difficulty in characterizing the amorphous phase. The current mechanochromic materials suffered from problems that low crystallinity, poor controllability, and low sensitivity. In the future, high-performance mechanochromic materials based on organic-metal compounds that can be used in sensors and anti-counterfeiting fields still need to be further explored. We expect that this paper can provide valuable reference for the development of mechanochromic materials.
